# The energy and water cycles under climate change

**DOI:** 10.1093/nsr/nwaa003

**Published:** 2020-01-16

**Authors:** Qingyun Duan, Anmin Duan

**Affiliations:** Qingyun Duan is a professor from Hohai University, and Anmin Duan is a professor from the Institute of Atmospheric Physics, Chinese Academy of Sciences

## Abstract

Energy and water cycles have been a hot research topic in the global scientific community. The global climate change observed over the last century is having a profound impact on global and regional energy and water cycles, leading to more frequent extreme climatic events and affecting water security, ecosystem and socioeconomic development around the world. The impact is especially obvious over the highland regions such as the Tibetan Plateau. Here we have an interview with one of world's most renowned experts in hydroclimatology, Professor Soroosh Sorooshian from the University of California at Irvine, to share his insights on the subject of energy and water cycles.

## ENERGY AND WATER CYCLES

**NSR:** What are the energy and water cycles?

**SS:** When the Earth is warmed by the energy received from the Sun, water in the ocean evaporates into the atmosphere, where it moves vertically and horizontally around the globe. When water vapor moves upwards, it cools and forms ice crystals that eventually fall as rainfall on the land surface, where part of it becomes runoff and flows into the ocean. The movement of water from the ocean to the atmosphere to the land and back to the ocean is called the water cycle. When water cycles through the Earth system, it accompanies the energy cycle as water moves and changes phases between liquid, vapor and solid (ice) by absorbing and releasing energy.

**NSR:** What is the role of the energy and water cycles in regional and global climate change?

**SS:** To understand the role of energy and water cycles in regional and global change, we need to first define some terminology. The first term is weather, which refers to the condition of the atmosphere we observe at a given time in a given place. Different aspects of weather we are familiar with include air temperature, rainfall, wind speed and humidity, etc. The second term is climate, which is defined as the averages and variations of weather conditions over several decades in a given place. Both weather and climate vary in space and in time, but over different time scales. While changes in weather can be seen instantly, climate change can only be observed over a long period of time. The ultimate driver of both weather and climate change is the energy received by the Earth from the Sun, which not only supports all forms of life on the Earth, but also governs the weather and climate conditions around the world through its redistribution in the five components of the Earth system: atmosphere, hydrosphere, cryosphere, lithosphere and biosphere. This redistribution of energy is what we call the energy cycle. As I mentioned earlier, the energy cycle always goes together with the water cycle. In a way, we can say that climate science is the study of the geophysical processes involved in energy and water cycles, a subject that is governed by the fundamental laws of physics. The energy and water cycle through the Earth system is very complex because it involves many different non-linear processes and many spatiotemporal scales, from minutes to millennial, from microscopic to global. This complexity is the reason why understanding and predicting the climate system is such a major challenge. It is also why the study of energy and water cycles has received a lot of attention in the scientific community. There are a number of international organizations and research programs that focus on this subject, including the World Climate Research Programme (WCRP), which is celebrating its 40th anniversary in 2019, and its four main projects namely: (i) Global Energy and Water cycle Exchanges (GEWEX); (ii) Climate and Ocean–Variability, Predictability, and Change (CLIVAR); (iii) Stratosphere–troposphere Processes And their Role in Climate (SPARC); and (iv) Climate and Cryosphere (CliC). In short, the understanding of energy and water cycles is critically important to the understanding of regional and global climate change.

**Figure:**
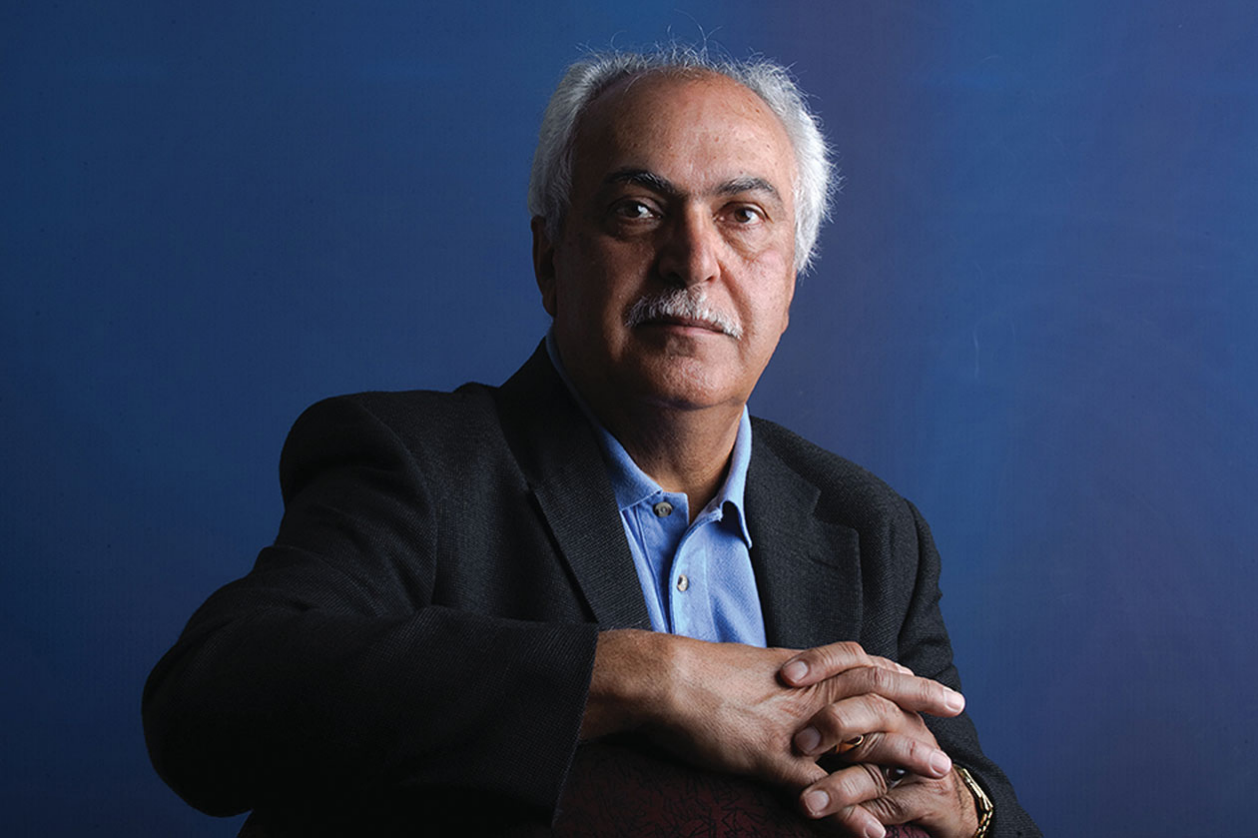
Dr Soroosh Sorooshian, Distinguished Professor of the University of California at Irvine and former Chairman of the Science Steering Group of the Global Energy and Water cycle Exchanges (GEWEX) project (*Courtesy of Dr Soroosh Sorooshian*).

GEWEX was initiated to coordinate science activities to facilitate research into the global water cycle and interactions between the land and the atmosphere.

—Soroosh Sorooshian

## GEWEX AND ITS CONTRIBUTION TO WCRP

**NSR:** As the former Chairman of the GEWEX Science Steering Group, can you explain to us what GEWEX is?

**SS:** I will start with giving a little history of the program. The idea for GEWEX was originated in the late 1980s. During that time, three senior scientists, Vern Suomi, Pierre Morel and Leonard Bengtsson, began the planning of a global study of water and energy cycles. This study, known as the ‘Global Energy and Water cycle Experiment’ in the beginning and recently changed to ‘Global Energy and Water cycle Exchanges’, was formally launched as a core project of WCRP in 1988. Dr Moustafa Chahine, who was also NASA’s Jet Propulsion Laboratory Chief Scientist, served as the founding Chair of the Science Steering Group (SSG). GEWEX was initiated to coordinate science activities to facilitate research into the global water cycle and interactions between the land and the atmosphere. The mission of GEWEX is ‘to measure and predict global and regional energy and water variations, trends, and extremes (such as heat waves, floods and droughts), through improved observations and modeling of land, atmosphere and their interactions; thereby providing the scientific underpinnings of climate services’. Water and energy are fundamental for life on Earth. Fresh water is a major pressure point for society owing to increasing demand and the vagaries of climate. Extremes of droughts, heat waves and wild fires as well as floods, heavy rains and intense storms increasingly threaten people's livelihoods and economic development as the climate changes. Other challenges exist on how clouds and aerosols affect energy and climate. The vision of GEWEX is therefore given as: ‘Better observations and analysis of these phenomena, and improving our ability to model and predict them, will contribute to increasing information needed by society and decision makers for future planning’.

**NSR:** How does GEWEX operate?

**SS:** GEWEX is organized into three research domains: modeling and prediction studies, hydrometeorology, and the analysis of global energy and water cycle variability. Those research activities are overseen by the GEWEX Science Steering Group (SSG) and coordinated by several GEWEX panels: the GEWEX Hydrometeorology Panel (GHP), the GEWEX Atmosphere System Studies (GASS) Panel, the GEWEX Land–Atmosphere System Studies (GLASS) Panel and the GEWEX Data Analysis Panel (GDAP).

The implementation of GEWEX evolved in three phases. Initially, GEWEX was created to take advantage of the new Earth observational satellites developed in the 1980s. Phase I (1990–2002) of GEWEX focused on developing analysis tools and models, using operational and research satellites, regional analyses of continental scale basins, and process studies to support the development of parameterizations of feedback processes (relating to clouds and land) for global climate models. The scientific exploitation of the data from the new satellite sensors began more aggressively in Phase II (2003–12). There were a number of evolutionary changes that occurred within GEWEX that set the stage for Phase III (2013–22). Building upon the results and experience from Phases I and II, new GEWEX Vision and Mission Statements were developed, and an Imperatives document that provides the activities necessary to accomplish these was created.

**NSR:** Can you be more specific about what the GEWEX Imperatives mean?

**SS:** Sure. The GEWEX Imperatives provide a detailed framework for GEWEX activities by focusing on seven areas where GEWEX can best advance water and energy cycle science. They were formed as GEWEX was undergoing a re-evaluation and capitalize on new perspectives and opportunities in the program. Those imperatives include six areas:

1. Data Sets: Foster development of climate data records of atmosphere, water, land, and energy-related quantities, including metadata and uncertainty estimates.
2. Analysis: Describe and analyze observed variations, trends, and extremes (such as heat waves, floods, and droughts) in water and energy related quantities.
3. Processes: Develop approaches to improve process-level understanding of energy and water cycles in support of improved land and atmosphere models.
4. Modeling: Improve global and regional simulations and predictions of precipitation, clouds, and land hydrology, and thus the entire climate system, through accelerated development of models of the land and atmosphere.
5. Applications: Attribute causes of variability, trends, and extremes, and determine the predictability of energy and water cycles on global and regional bases in collaboration with the wider WCRP community.
6. Technology Transfer: Develop new observations, models, diagnostic tools and methods, data management, and other research products for multiple uses and transition to operational applications in partnership with climate and hydrometeorological service providers.

**NSR:** What are the major contributions of GEWEX to WCRP?

**SS:** GEWEX contributes to WCRP by addressing the WCRP Grand Science Challenges, which were developed by the WCRP Joint Scientific Committee (JSC) through consultation with WCRP sponsors, stakeholders and affiliate networks of scientists. WCRP promotes the Grand Challenges through community-organized workshops, conferences and strategic planning meetings to identify exciting and high-priority research that requires international partnership and coordination, and that yields ‘actionable information’ for decision makers. The current WCRP Grand Challenges are:

1. Melting Ice and Global Consequences
2. Clouds, Circulation and Climate Sensitivity
3. Carbon Feedbacks in the Climate System
4. Weather and Climate Extremes
5. Water for the Food Baskets of the World
6. Regional Sea-Level Change and Coastal Impacts
7. Near-term Climate Prediction

In response to the WCRP Grand Challenge initiative, GEWEX has developed four science questions to be addressed in the next 5–10 years. GEWEX Science Questions (GSQs) address the contributions that water and energy cycle science can bring to society in four major areas. They examine problems such as our understanding of precipitation variability, changing water availability and hydrologic extremes such as drought and floods. The GSQs were developed by GEWEX’s Scientific Steering Group as a contribution to the WCRP’s Grand Challenges. By posing these questions and their related issues, GEWEX has created a framework for its current and future efforts in the next 5–10 years. The GSQs are outlined below:

1. Observations and Predictions of Precipitation: How can we better understand and predict precipitation variability and changes?
2. Global Water Resource Systems: How do changes in land surface and hydrology influence past and future changes in water availability and security?
3. Changes in Extremes: How does a warming world affect climate extremes, esp. droughts, floods, and heat waves, and how do land area processes, in particular, contribute?
4. Water and Energy Cycles and Processes: How can understanding of the effects and uncertainties of water and energy exchanges in the current and changing climate be improved and conveyed?

## ROLE OF THE TIBETAN PLATEAU

**NSR:** Can you tell us the significance of energy and water exchanges over elevated highlands?

**SS:** Energy and water exchanges over elevated highlands have a significant impact on climate. High mountains affect climate by blocking and redirecting airflow and altering atmospheric circulation patterns. As air mass is forced over steep slopes, it cools down. The higher the elevation above sea level, the colder the air mass gets. Air mass also becomes thinner and water vapor carried in it has less capacity to absorb and retain heat and thus becomes ice crystals that eventually fall as rain or snow. Some of the rain or snow evaporates before reaching the ground. Mountains also generate other local effects such as drag and tunnel effects from the change of air pressure and flow, resulting in strong winds and violent weather formations. The mountain formation increases the total land area covered by snow and ice caps, which results in more reflection of sunlight into the atmosphere, and reduces the overall amount of energy absorbed at the Earth's surface.

**NSR:** When we talk about elevated highlands, the Tibetan Plateau (TP) stands out. Can you shed some light on how TP affects global and regional energy and water cycles?

The Asian monsoon is the consequence of the seasonal change not only in land–sea thermal contrast but also in the thermal forcing of large-scale mountains.

—Soroosh Sorooshian

**SS:** Indeed, Tibetan Plateau (TP) is a unique case not like any other on our planet when talking about elevated highlands. With its massive spatial size equivalent of 1/6 of Asia's land area and its average elevation over 4000 m, TP has been described as the ‘roof of the world’ and referred to by the scientific community as the ‘third pole’. On this elevated terrain there is also a large number of intersecting isentropic surfaces in the lower troposphere. Along its sloping surfaces, the cooling in winter causes the near-surface air to slide downward and diverge toward its surroundings, whereas the surface heating of the slope in summer results in near-surface air ascent, causing the surrounding air to converge toward the plateau. More significantly, due to its huge size, the surface-sensible heating of TP produces a large-scale surface cyclonic circulation and works as an immense sensible-heat-driven air pump (SHAP), which transports abundant water vapor from ocean to land to support the Asian continental monsoon. In addition, the plateau's heating produces a subtropical monsoonal meridional circulation and creates a large-scale air ascent background in subtropical Asia. Therefore, the Asian monsoon is the consequence of the seasonal change not only in land–sea thermal contrast but also in the thermal forcing of large-scale mountains. Overall, TP’s elevated heating/cooling together with mechanical forcing not only plays a dominant role in monsoon circulations and regional energy and water cycles over Asia, but also exerts enormous influence on atmospheric circulations and climate over Europe and North America, and atmosphere–ocean interactions in the tropics and mid-latitudes of the North Pacific though teleconnections.

**NSR:** TP plays a critical role in water security in Asia. Can you elaborate a little bit on the role of TP in Asia's water security, especially in the context of climate change?

**SS:** From a hydrological perspective, TP is regarded as the ‘water-tower’ of Asia, feeding water into several major river basins, including the Yangtze River, Yellow River, Yalung Zapo River/Brahmaputra River, Lan-Tsang Chiang/Mekong River, among others. TP and the surrounding mountain ranges contain 36 800 glaciers. Glaciers, ice and snow cover about 17% of the Himalayan area, with glaciers occupying a total area of ∼50 000 km^2^. It also has the largest concentration of inland lakes in the world with a total lake surface area of ∼37 000 km^2^.

The warming of the planet Earth over the last 50 years has had a profound impact on the water resources and hydrologic cycle over TP. Significant changes have been observed in the space–time distribution of water resources, especially in the cryosphere. Glaciers have been retreating, melting of the snow and glaciers has been accelerating, and the snow season has been shrinking. The changing cryosphere has resulted in an increase in the sizes of many inland lakes. Meanwhile, there are significant changes in climate extreme indices in the region as well, especially with respect to extremes of surface air temperature. Those changes have deep implications for ecosystem protection and sustainable socio-economic development in the region. They may also have adverse effects on the streamflow in the lower reaches of the major rivers that originate from TP.

**NSR:** Can you tell us the importance of investigating the land–atmosphere coupling over the Tibetan Plateau and its impact on regional and global climate prediction?

**SS:** As I have already discussed, TP, as the most prominent and complicated land formation on the planet, exerts a tremendous influence on regional and global climate. Previous research has established that TP is a region with the strongest land–atmosphere interactions in the mid-latitude areas. However, there is still a lack of quantitative understanding of the interactions between the land surface and atmosphere. This knowledge gap makes it difficult to understand and predict the complete energy and water cycles over TP and their effects on global climate change. Some of the urgent science questions and issues identified by the scientific community that we need to address include the following. (i) Can we develop better parameterization schemes to represent the complicated land surface heat and water processes over TP? (ii) What is the structure of the boundary layer and how does it vary spatially over TP? (iii) What is the radiative effect of the surface albedo on energy and water cycles and how do those effects vary spatially over TP? (iv) Can we quantify the regional and global weather and climate effects of the land–atmosphere interaction over TP? Answers to those science questions can go a long way to improve our understanding of the critical role of land–atmosphere coupling over TP in regional and global climate change.

## CHALLENGES AND MORE

**NSR:** What are the challenges we are facing in the investigation of the energy and water cycles?

**SS:** My expertise is limited to hydroclimatological aspects and in this regard, there are a number of challenges we face in the study of global energy and water cycles. The foremost challenge is the observations and predictions of hydroclimatic elements involved in the energy and water cycle, which are fundamental to understanding and predicting global and regional climate change. Current observational systems suffer from various limitations. The traditional observations based on *in situ* and ground-based gauging stations have sparse spatial coverage and cannot meet the need required by land surface models. This is especially true in remote areas such as TP, as well as in many developing countries. Satellite data have become increasingly available over the last 30 years and have shown promise for modeling applications. Available remote sensing observations now include precipitation, soil moisture, water storage and land surface characteristics. The recently available Global Precipitation Mission (GPM) represents a step forward in improving precipitation estimates. Other missions include Soil Moisture Active Passive (SMAP) and Soil Moisture and Ocean Salinity (SMOS) to measure and map Earth's soil moisture and freeze/thaw state to better understand terrestrial water, carbon and energy cycles and to provide new insights into the Earth's water cycle and climate. I understand that China has also launched a series of Fengyun meteorological satellites, which are part of the global satellite constellation.

For climate applications, the climate data record (CDR) of NOAA in the United States has produced a number of remotely sensed data sets that are freely available to the scientific community at large. One such product, known as PERSIANN-CDR (Precipitation Estimation from Remotely Sensed Information using Artificial Neural Networks–Climate Data Record) is a high-resolution (daily at 0.25°) precipitation data set with global coverage of 60°N to 60°S and data duration of more than 35 years. This product is developed by our Center for Hydrometeorology and Remote Sensing (CHRS) at the University of California, Irvine (UCI). It is designed to address the need for studying trends and extreme events, and has proven to be valuable especially for studying TP where *in situ* precipitation observations are very scarce.

I wish to emphasize that the rapid progress in dealing with highly complex problems such as the climate system would not have reached the level of advances we see today without international collaboration.

—Soroosh Sorooshian

**NSR:** What are the other challenges?

**SS:** The second challenge is to understand the impact of anthropogenic activities on energy and water cycles. With increasing world population and economic development, the influence of human activities on energy and water cycles is becoming more and more evident. Many of our models of energy and water cycles are based on our understanding of the natural systems, which cannot be applied directly in the real-world systems where human beings have a significant influence. There is a need to develop models that can better represent the complex human–Earth system interactions. In particular, special attention is needed to more realistically represent the complex interaction between the land surface and all anthropogenic effects. This encompasses all aspects of global change, including water management, land use change and urbanization. The ecosystem response to climate variability and responsive vegetation must be included, as must cryospheric changes such as permafrost thawing and changes in mountain glaciers. Feedbacks, tipping points and extremes are of particular concern. The results should enhance the evaluation of the vulnerability of water systems, especially to extremes, which are vital for considerations of water security and can be used to increase resilience through good management and governance.

The third challenge is to understand climatic extremes under a changing world. There is plenty of evidence indicating that anthropogenic activities are the cause of climate warming observed over the last century. A warming world is expected to increase the occurrence and magnitude of extremes such as droughts, heavy storms, floods and heat waves, as well as the space and time distributions of rain and snow. Such changes are related to an acceleration of the hydrologic cycle and circulation changes, and include the direct impact of warmer conditions on atmospheric water vapor amounts, rainfall intensity and snow-to-rain occurrence. How well are models able to handle extremes and how can we improve their capability? New improved and updated data sets at high frequency (e.g. hourly) are needed to properly characterize many of these facets of our climate and to allow for assessment against comparable model data sets. New activities are needed to promote analyses quantifying which changes are consistent with our expectations and how we can best contribute to improving their prediction in a future climate. Confronting models with new observationally based products will lead to new metrics of performance and highlight shortcomings and developmental needs that will focus field programs, process studies, numerical experimentation and model development. New applications should be developed for improved tracking and warning systems, and assessing changes in risk of drought, floods, river flow, storms, coastal sea level surges and ocean waves.

**NSR:** Do you have any other thoughts to be shared with the NSR audience?

**SS:** Thank you for asking. As my final remarks, based on my nearly 30 years of involvement with international programs such as GEWEX and WCRP, I wish to emphasize that the rapid progress in dealing with highly complex problems such as the climate system would not have reached the level of advances we see today without international collaboration. There has been a truly remarkable evolutionary process comparing the early days with the present time in terms of scientists from across the globe working together and publishing their results related to many of the water and energy cycle issues and challenges I discussed above. In particular, the engagement of the Chinese scientific community and the strong support provided for their research by various Chinese funding agencies, particularly the National Natural Science Foundation of China (NSFC) has been unmatched, and much appreciated by the international programs.

Furthermore, among the many scientists who have worked on gaining understanding of global energy and water cycles, especially the role of TP on the hydrological cycle and the Asian monsoon system, I must mention the seminal contributions of Chinese hydrological and climate scientists/modelers. Among them are Academician Professor Guoxiong Wu, who has been one of the early players in the GEWEX and WCRP program, and Academician Professor Tandong Yao, who, along with renowned scientists Lonnie Thompson and Volker Mosbrugger, founded the Third Pole Environment (TPE) international program, which has been endorsed by UNESCO (United Nations Educational, Scientific and Cultural Organization) as its flagship program and has been in close partnership with UNEP (United Nations Environment Programme) and WMO (World Meteorological Organization). It is through these strong collaborative efforts and unselfish contributions of the scientists that I foresee the unraveling of many of the key questions in the coming decade.

